# Implementing virtual diabetes self-management education in two health systems (VIDA): a mixed-methods pilot study

**DOI:** 10.3389/fcdhc.2026.1784495

**Published:** 2026-06-26

**Authors:** Manuel Panzardi, Andrea Gomez, Courtney Borsuk, Abigail M. Durgan, Erin M. Staab, Nikita C. Thomas, Chasity Kasir, Wen Wan, Michael Quinn, Iridian A. Guzman, Morgan Lindsey, Claudia P. Garcia, Kenyetta Sims, Amanda Campbell, Cynthia T. Schaefer, Danielle Lazar, Arshiya A. Baig

**Affiliations:** 1 The University of Chicago Pritzker School of Medicine, University of Chicago, Chicago, IL, United States; 2 Department of Medicine, University of Chicago, Chicago, IL, United States; 3 Advocate Health, Milwaukee, WI, United States; 4 Access Community Health Network, Chicago, IL, United States; 5 Midwest Clinicians’ Network, East Lansing, MI, United States

**Keywords:** chronic disease education, diabetes self management education, group education, SMART goal-setting, telehealth

## Abstract

**Introduction:**

Group-based diabetes self-management education (DSME) has long been shown to improve health outcomes, but how to effectively adapt these programs to virtual settings in diverse healthcare systems remains unclear. Utilizing the RE-AIM implementation framework, this mixed-methods pilot study evaluated the feasibility, benefits, and challenges of implementing virtual group DSME for adults with Type 2 diabetes mellitus in two distinct systems: a federally qualified health center and a private, not-for-profit health system.

**Methods:**

Adults with elevated glycosylated hemoglobin (A1c) levels and at least one cardiovascular comorbidity were invited to participate in six monthly 60- to 90-minute online group education sessions led by diabetes educators. Sessions focused on disease and lifestyle education, peer support, and structured goal setting. Quantitative data captured patient recruitment, attendance, and engagement metrics. Semi-structured interviews with patients, providers, and staff explored perceived benefits, barriers, and implementation experiences.

**Results:**

Of 97 eligible patients, 50 were reached and 11 enrolled, with 10 completing at least one session. Participants had an average age of 63.5 years old, were 90% African American and 80% female, and had an average A1c of 8.7% (SD 0.7). The average attendance rate was 3.6 (SD 1.9) sessions. The most frequent reasons for declining enrollment in the study were competing responsibilities and stating they did not need additional diabetes education, while accessing technology was rarely a limiting factor. Participants reported high satisfaction with the program, emphasizing the value of shared experiences, increased accountability, improved disease knowledge, and convenience of attending virtually from home. Providers and staff viewed the sessions as a valuable adjunct to clinical care that reinforced behavioral goals beyond the limits of brief primary care visits. Reported challenges included scheduling conflicts with patients and mistrust in medical advice. The intervention demonstrated strong fidelity to implementing the treatment design and curriculum, with most patients successfully formulating goals related to exercise and blood glucose monitoring.

**Discussion:**

This study provides initial evidence that virtual group DSME can be feasible, well-received, and adaptable across different healthcare environments. It provides initial evidence in understanding how technology-enabled group models can deliver chronic disease education in different health system contexts while empowering self-management in diverse urban populations.

## Background

Diabetes self-management education (DSME) is essential for the comprehensive care of diabetes. It has been shown to improve glycemic control, enhance self-efficacy, and reduce healthcare utilization (1, 2). DSME programs provide individuals with the knowledge and confidence to manage their diabetes and related comorbidities (3). Although DSME is a cornerstone of effective diabetes care, national data show that less than 5% of Medicare beneficiaries with diabetes and 6.8% of privately insured adults with diabetes have received these services (4). DSME group-based formats are recognized as a cost-effective and efficient way to deliver this education (1, 5). Group-based DSME fosters peer learning and psychosocial support while addressing common barriers to individual education, such as limited provider time and access to certified educators (6, 7).

During the COVID-19 pandemic, health systems adapted out of necessity by shifting in-person DSME programs into virtual formats (8, 9). Telemedicine has now become a crucial part of chronic disease management and is expected to become more common in the future (10). While virtual DSME has the potential to expand access, particularly for patients facing transportation, mobility, or work-related barriers, its implementation and effectiveness in real world clinical settings remain understudied (11–14). Existing literature highlights challenges related to technology access, digital literacy, cost, and resistance to change in clinical practice as barriers to implementation (12, 15–17). However, the Diabetes Learning in a Virtual Environment (LIVE) study, a multisite randomized controlled trial of a virtual interactive education environment with synchronous classes and peer support, demonstrated feasibility and improvements in self-management behaviors, supporting the potential of virtual DSME delivery (18). Few studies have examined how virtual DSME programs can be implemented across distinct health systems serving diverse populations.

The VIDA (**Vi**rtual **D**iabetes Group Visits **A**cross Health Systems) project was designed to address these gaps by piloting a virtual group DSME model in two health systems, one federally qualified health center (FQHC) site and one private health system clinic. The aims of this pilot study were to evaluate implementation outcomes in terms of number of and barriers to enrollment and attendance, and to assess the fidelity of virtual group-based DSME delivery in two health systems. For the qualitative component of this study, the aims were to determine feasibility and acceptability of GVs through patient and care team interviews. This mixed-methods evaluation of virtual group-based DSME in two distinct health systems will highlight opportunities to improve broader implementation of this healthcare delivery model.

## Methods

### Setting

The pilot study was conducted as a partnership between the University of Chicago, Midwest Clinicians’ Network (MWCN), and two health systems: a FQHC network and a large, private, not-for-profit health system. The VIDA intervention was piloted at one FQHC site and one private site. The FQHC network primarily provides primary care and has 36 sites across the Chicago metropolitan area. At the pilot FQHC site, 37% of patients were Black/African-American, 33% Hispanic/Latino, and 13% White. Of their patients, 13% had T2DM and 17% of these patients had a glycosylated hemoglobin (A1c) > 8%. The private clinic belongs to a large, diverse, integrated private, not-for-profit health system, providing care for more than 129 primary care clinics in Illinois with access to subspecialty care. At the pilot private clinic, 48% of patients were Black/African-American, 3% Hispanic/Latino, and 38% White. Of their patients, 18% had T2DM and 7% of these patients had an A1c > 8%. In collaboration with health system partners, each site was chosen based on the burden of diabetes and need for self-management education in the community, previous experience with research studies, and representation of broader health system characteristics. The study was approved by the University of Chicago IRB. Both participating health systems entered into IRB reliance agreements with oversight by the University of Chicago IRB. To aid implementation, biweekly meetings with members from both healthcare systems and the study team were conducted over the duration of the project. These meetings were performed to equitably collaborate on study and intervention design, curriculum formation, and to determine relevant measurable endpoints for data analysis.

### Participants

In order to be eligible for the program, patients were required to have at least one visit at a participating clinic in the year prior to the first session. Other inclusion criteria included a Type 2 diabetes diagnosis, age greater than or equal to 18 years old, English speaking, A1c greater than 8% within 6 months prior to the first session, and at least one additional cardiovascular condition. Eligible cardiovascular conditions were defined as a history of hypertension, heart disease, stroke, hyperlipidemia, peripheral vascular disease (PVD), or body mass index (BMI) greater than or equal to 30 kg/m^2^. Patients with higher A1cs were prioritized in the recruitment process. All enrolled patients provided written informed consent.

Each site aimed to recruit 5–7 patients for the virtual group-based DSME sessions. The University of Chicago and health system research teams attended staff meetings with primary care providers (PCPs) and clinical staff at each intervention site to inform them of the study objectives and virtual group-based DSMEas an option for their patients. Providers and staff could refer patients by contacting the health systems’ project coordinators or diabetes educators. Data analytics teams at each site also extracted lists of patients who met eligibility criteria. Project coordinators asked PCPs for assent to contact patients about the study then sent MyChart messages, called, texted, or asked PCPs for help reaching eligible patients. The project coordinators or diabetes educators attempted initial contact with patients up to three times by each method (e.g., 3 MyChart messages, 3 phone calls).

### Conceptual framework

The planning and evaluation of this intervention was informed by the RE-AIM framework, a widely utilized implementation framework (19). Key RE-AIM dimensions (Reach, Effectiveness, Adoption, Implementation, and Maintenance) were incorporated into the planning and evaluation of the intervention (19). Reach, the representativeness of participating individuals, was quantitatively evaluated by measuring the demographic and background characteristics of participants who participated compared to non-participants. Effectiveness, impact on intended outcomes, was assessed with qualitative analysis of interviews of patients and providers who commented on the strengths and weaknesses of the program. Assessing the scalability of this model was essential to the overall design of the project and the two sites were carefully selected to be representative of each respective health system’s characteristics. Implementation variables (including but not limited to attendance patterns, fidelity to protocol checklists, and modifications made by diabetes educators) were tracked by project coordinators. Maintenance of the individual behaviors in the long-term was not directly assessed in this pilot study, but will be assessed in an upcoming randomized control trial of this program.

### Intervention

Structured virtual group-based DSME education consisted of six monthly sessions lasting 60 to 90 minutes in duration. Sessions were delivered on an online conferencing platform. Each session had core components: diabetes education, group social support, and goal setting. Diabetes education was organized into six, specialized curricula delivered at each session. Curriculum topics included: Introduction to Diabetes, Nutrition, Stress Management, Diabetic Medications, Physical Activity, and Overview/Summary. The curricula were developed collaboratively by the University of Chicago, FQHC, and private health system study team members. Diabetes educators were trained using the shared curriculum and standardized session structure to promote consistency across sites. Fidelity was monitored through review of session delivery, including minute-by-minute tallies of time spent on each slide to assess adherence to planned content and session flow. During the team biweekly meetings, any deviations were discussed.

Virtual sessions were led by a diabetes educator hired by each health system and trained by the study team and health system research team. The educator’s role was to provide information about diabetes, encourage patients to set Specific, Measurable, Achievable, Relevant, and Time-bound (SMART) goals for diabetes self-management, empower them to take control of their disease, and make healthy lifestyle changes. Patients were asked to create a SMART goal at each session related to the session’s focused topic (e.g., nutrition, stress management, diabetes management, physical activity). Patients were provided with information on how to access established virtual video platforms used by each healthcare system. Patients could choose to attend sessions via phone only if video was not possible.

Patients were recommended to continue visiting their PCP quarterly per American Diabetes Association guidelines (20). PCPs received progress notes from the diabetes educators after each session and were notified when patients initially enrolled, attended, or were absent from sessions. Patients were given a binder with curriculum, educational materials, handouts, and a bag with self-management related items (e.g., pedometer, cutting board, measuring cups, pill separator, pen and notebook).

### Data collection

#### Recruitment, enrollment and engagement

Project coordinators at both sites tracked recruitment, attendance, and information related to patient access of virtual platforms. The study team requested a patient recruitment dataset that included methods by which patients were reached, which patients expressed interest, which patients were enrolled, and reasons for no contact or refusing to participate. Outcomes of interest for recruitment and enrollment were the number of eligible, contacted, and enrolled patients as well as reasons for declining participation. Demographic information from this data set was collected to compare participant versus non-participant characteristics for age, sex/gender, race/ethnicity, health insurance, primary language, most recent A1c, and other cardiovascular comorbidities. Attendance and participation data was tracked to evaluate engagement with the program as defined by average session attendance per patient, average group size per session, and percentage of sessions where patients attended with cameras on. This data was also collected to evaluate for significant differences in attendance based on health system or demographic characteristics. Patient priorities were evaluated based on the percentage of notes from project coordinators which described a particular theme discussed with patients in their SMART goals or reported challenges. These notes were collected during each session and uploaded for University of Chicago study team analysis using descriptive statistics. Lastly, benefits and challenges of the intervention were evaluated based on thematic patterns identified during qualitative analysis of interviews with patients, providers, and study staff.

#### Interviews

Patients, diabetes educators, project coordinators, and PCPs with patients enrolled in the intervention were invited to participate in 30-minute, 1:1 semi-structured interviews. Interviews were conducted via online conferencing platforms without video or via telephone after 3-months of virtual group-based DSME sessions being implemented. Interviews were led by trained UChicago and MWCN staff members. Participants were asked to describe what they liked/disliked, what they found helpful/unhelpful for managing diabetes, and what they found to be beneficial or challenging about the sessions. Some example questions were: “What did you think of the materials you received?” and “What changes or improvements would you recommend?” Interviews were audio-recorded for data analysis and were then transcribed professionally. The interviews were conducted with a goal of theme saturation but were limited by the small number of patient and staff respondents.

### Analysis

#### Quantitative data analysis

The quantitative analysis aimed to characterize recruitment, enrollment, attendance, and engagement patterns and to identify barriers to participation. Project coordinators uploaded recruitment, enrollment, attendance, and engagement data to a third-party data broker to securely merge and standardize data before sharing with the University of Chicago study team. Following data cleaning, descriptive analyses were conducted in Excel and RStudio. Enrollment metrics included the number of patients identified as eligible, contacted, and enrolled in the intervention. The frequency of reported reasons for declining participation was also analyzed to identify barriers to enrollment. Demographic and clinical characteristics of eligible patients, including age, sex, race, ethnicity, insurance status, cardiovascular comorbidities, and baseline hemoglobin A1c, were compared among enrolled participants, individuals who declined participation, and those who could not be contacted.

Exploratory statistical analyses included variable transformation, Fisher’s exact tests, and Wilcoxon rank-sum tests. Because the primary purpose of this pilot study was to assess feasibility and inform the design of a subsequent randomized controlled trial, a formal sample size calculation was not performed for these exploratory analyses, and the study was not powered to detect statistically significant differences between groups.

#### Qualitative analysis of interviews

Four investigators used a modified template approach to text analysis using the interview guide to create an initial codebook. As investigators reviewed the interviews, statements were categorized into recurring thematic domains identified inductively from the data. Themes were compiled and frequency counts were calculated in Microsoft Excel. Each transcript was assigned to two coders, with a combination of coder pairings. Each member coded the assigned transcript independently then met with the second coder to discuss agreement. The coders met to expand on themes and finalize the codebook, and discrepancies were resolved through discussion and consensus until agreement was reached. Formal intercoder reliability statistics were not calculated.

In the context of this pilot study, where patient goal-setting behavior was being evaluated in a real-world educational setting and participants were learning to make SMART goals, we used a modified definition of SMART goals. Standard SMART goals must meet all five criteria. In this pilot study, patient goals were considered SMART aligned in the analysis if they met at least one of the five criteria (Specific, Measurable, Achievable, Relevant, and Time-bound). This approach allowed us to assess progression in goal-setting skills even when patients did not fully develop a SMART goal meeting all five criteria.

During the sessions, project coordinators recorded observer-documented notes that included patient behaviors and comments related to diabetes self-management, and overall session observations. These notes were categorized into several categories including diabetes management education, patient-provider interactions, monitoring diabetes indicators, exercise goals, nutrition goals, speaking habits, exercise challenges, and nutrition challenges. For this analysis, only patient statements from these coordinator-recorded notes were included (N = 61), excluding the session observation notes.

## Results

### Recruitment and enrollment

**Figure 1:**
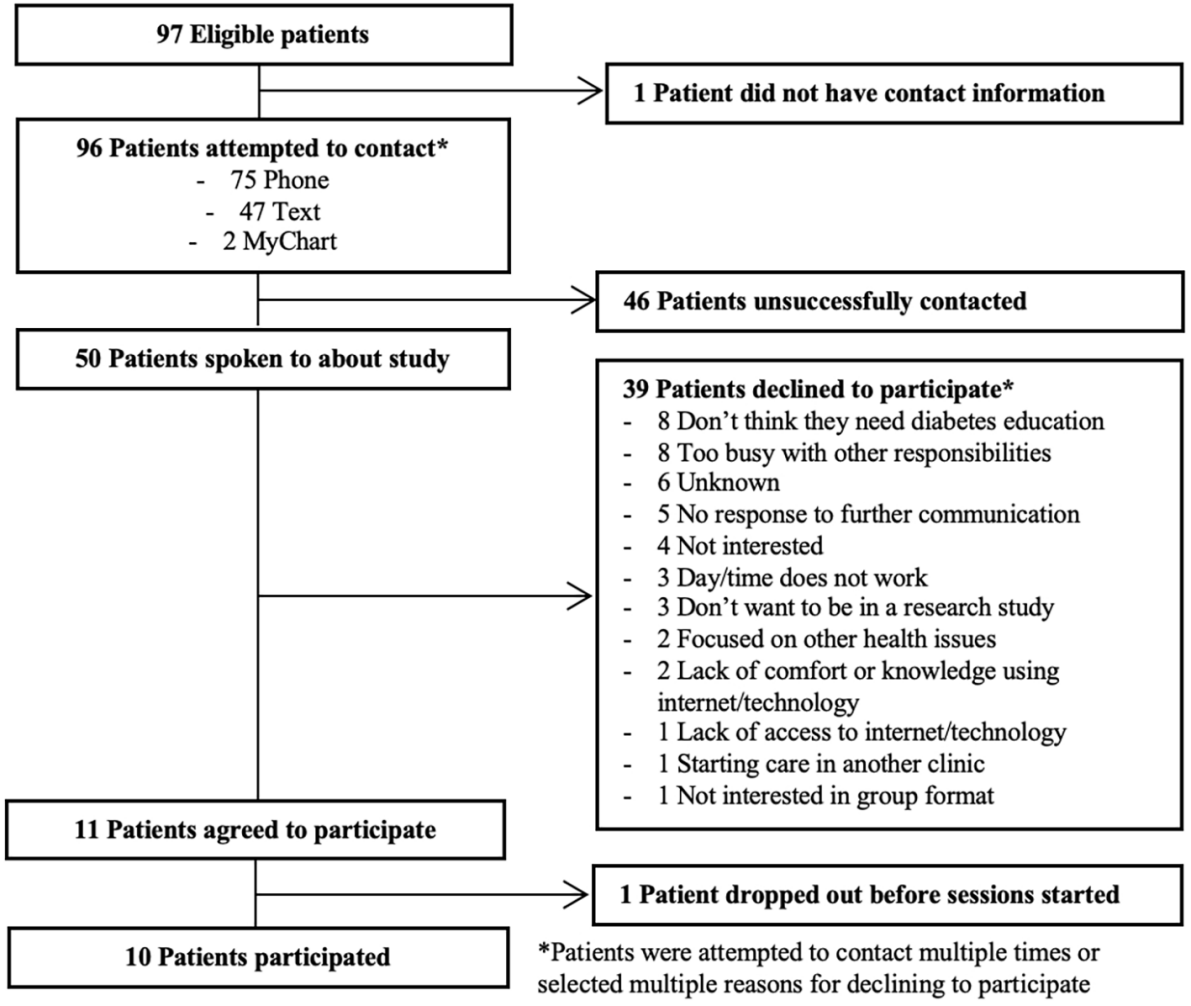
Enrollment flow diagram.

Of the 97 patients identified as eligible for the study, project coordinators attempted to contact 96 patients. The enrollment rate was 22%, with 11 patients agreeing to participate out of 50 patients who were successfully contacted. One patient dropped out prior to the group sessions, leaving 10 patients in the study. **Figure 1** summarizes the pattern of enrollment. Among the 39 patients who were successfully contacted but declined to participate, the most common reasons for declining participation were: “busy with other responsibilities” (21%, *n* = 8) and “do not think they need diabetes education” (21%, *n* = 8). Neither lack of comfort with (5%, *n* = 2) nor access (3%, *n* = 1) to internet/technology were reported as significant barriers to participation. 11 patients (28%) either did not respond to additional communication or did not provide a reason for declining participation.

### Demographics

A total of 97 patients were identified for recruitment across both pilot sites. Demographic information is summarized in **Table 1**. Participants who enrolled were similar in age, ethnicity, insurance status, and baseline A1c to those who declined enrollment. Sex distribution differed modestly, with a higher proportion of women among enrolled participants (80%) compared with those who declined (53%) or were unable to be contacted (57%), although this difference did not reach significance on Fischer’s exact testing (p > 0.05). Enrolled participants were also more likely to be African-American/Black (90%) than those who declined (65%) or were unable to be contacted (66%). Formal comparisons of baseline demographic characteristics showed no significant differences between enrolled and non-enrolled patients.

**Table 1:** Demographics of participant and non-participants.

Characteristic	Enrolled (N = 10)	Declined Enrollment (N = 40)	Unable to Contact (N = 47)
Age, Mean (SD)	63.5 (8.86)	62.53 (11.41)	64.40 (12.32)
Female, N (%)	8 (80%)	21 (53%)	27 (57%)
Male, N (%)	2 (20%)	19 (47%)	20 (43%)
Race, N (%)			
African-American/Black	9 (90%)	26 (65%)	31 (66%)
White	1 (10%)	9 (23%)	11 (23%)
Other	0 (0%)	5 (13%)	5 (11%)
Ethnicity, N (%)			
Non-Hispanic/Latino	9 (90%)	39 (97%)	45 (96%)
Hispanic/Latino	1 (10%)	1 (3%)	2 (4%)
Insurance, N (%)			
Medicaid	3 (30%)	12 (30%)	12 (26%)
Medicare	5 (50%)	16 (40%)	25 (53%)
Private	1 (10%)	9 (23%)	10 (21%)
Uninsured/Self-Pay	1 (10%)	3 (8%)	0 (0%)
CV Comorbidities, N (%)			
Hypertension	9 (90%)	36 (90%)	39 (83%)
Stroke	0 (0%)	3 (8%)	3 (6%)
Hyperlipidemia	7 (70%)	22 (55%)	29 (62%)
PVD	2 (20%)	6 (15%)	6 (13%)
BMI > 30	4 (40%)	16 (40%)	22 (47%)
Recruitment A1c, Mean (Range)	8.74 (8.1 – 9.8)	9.10 (8.1 – 11.8)	8.58 (8.1 – 9.7)
Diabetes Medications			
Metformin, N (%)	6 (60%)		
Sulfonylurea, N (%)	5 (50%)		
SGLT2 inhibitor, N (%)	4 (40%)		
GLP1 agonist, N (%)	3 (30%)		
Insulin, N (%)	3 (30%)		
DPP-4 inhibitor, N (%)	1 (10%)		

Among the 10 enrolled patients, the average age was 63.5 (SD 8.9) and mean A1c was 8.7 (range 8.1 - 9.8). Patients were 80% female, 90% Black/African-American, and 10% Hispanic/Latino. Six of the enrolled patients were from the private health system clinic and four were from the FQHC site. Across all groups, the majority of patients utilized Medicaid or Medicare. The most common diabetes medication taken by enrolled patients was Metformin (60%) and 30% of enrolled patients were using insulin.

Private health system clinic patients had more average combined appointments and phone calls with PCPs in the year prior to the first session (8.7 private *vs*. 7.8 FQHC). Private health system clinic patients had, on average, 4.0 combined appointments/phone calls with an endocrinology specialist in the year prior to the first session. No FQHC patients had a documented visit with an endocrinology specialist in the year prior to the first session.

### Attendance, participation, and health system engagement

All patients attended, on average, 3.6 (SD 1.9) sessions. In the FQHC site, the four patients attended 3.3 sessions on average, while the six private clinic patients attended, on average, 3.8 sessions. The average group size per session was 2.2 patients in the FQHC site and 3.8 patients in the private clinic. Only one patient did not attend any sessions. Higher attendance at sessions was not significantly associated with sex, study site, ethnicity, or CV comorbidities (*p*-values ranged from 0.4 – 1.0).

The private clinic had more patients engage with sessions with their camera on relative to the FQHC site (56% private *vs*. 42% FQHC). Both systems had a similar proportion of patients log on to the conferencing platform with their computer (65% private *vs*. 62% FQHC) versus their phone (35% private *vs*. 38% FQHC). Neither of these differences were significant on Fischer’s exact testing.

### SMART goal setting and patient priorities

Patients were able to engage in goal setting during sessions by creating SMART goals which project coordinators recorded and followed up with a subsequent sessions. Initially not every goal made by patients met the five elements of SMART, as patients were still learning how to structure SMART goals. Across both cohorts and all six sessions, the nine patients who attended any session set a total of 23 SMART goals. Nine (39%) goals were completed.

**Figure 2:**
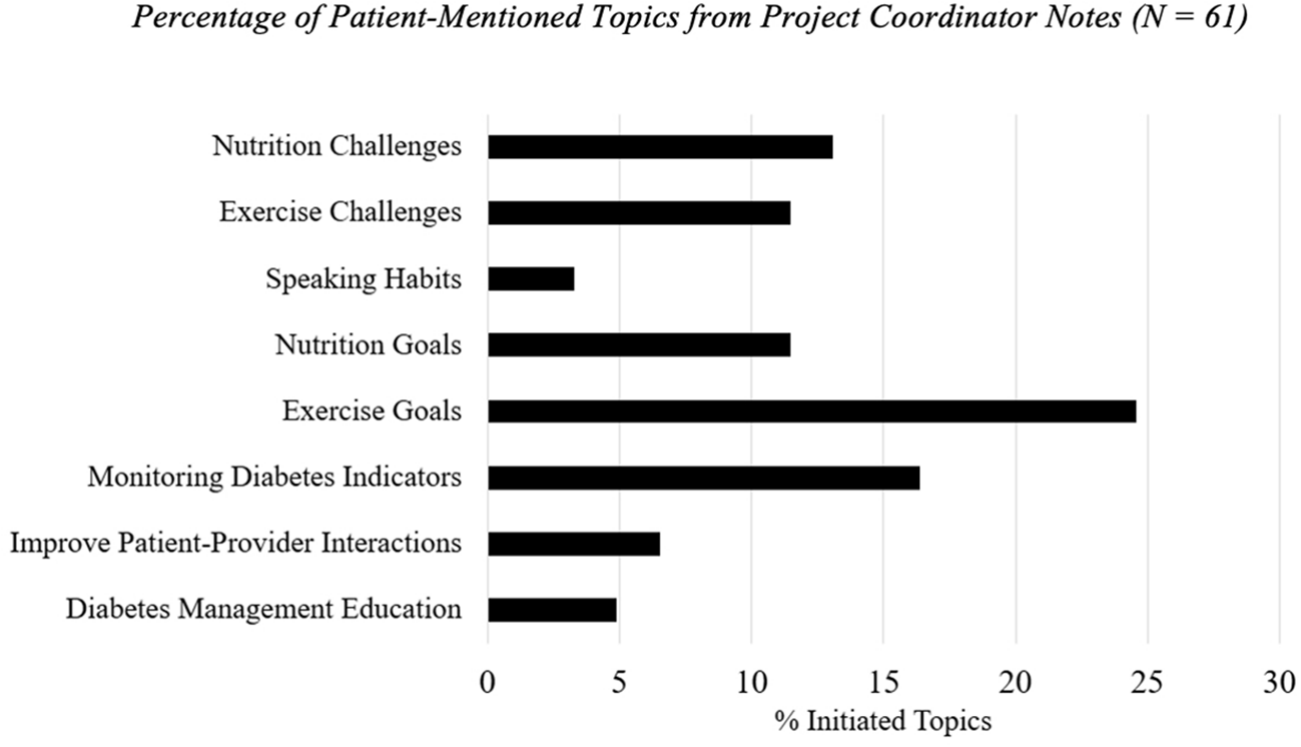
Percentage of patient-mentioned topics from project coordinator notes (N = 61).

Across the six sessions, the qualitative analysis of project coordinator notes on patient behaviors (N = 61) indicated that exercise goals were the primary goal focus, followed by goals related to monitoring diabetes indicators (i.e, A1c) (**Figure 2**). Nutrition challenges were mentioned slightly more than exercise challenges, although exercise goals were spoken about more frequently. Some participants wanted to speak more positively and decrease the amount of “negative talk” in their week, and the frequency of this topic is represented by Speaking Habits in **Figure 2**.

During the second session focused on nutrition, one patient’s SMART aligned goal was: *“…to keep a detailed food diary using the provided notebook for food diary kept with her at all times for 5 days to start to help keep a tally of foods eating good or bad for tracking blood glucose.”* When reminded of the criteria during the third n session, all patients set developed SMART aligned goals. That session focused on stress management, and one participant’s goal was: *“…to track and monitor blood glucose readings using freestyle meter for 2 weeks by checking meter readings 3–4 times daily to help manage blood glucose levels.”* Approximately half of patients set SMART aligned goals in the fourth and fifth sessions.

### Benefits and challenges

#### Benefits of structured virtual group-based DSME

##### Overall experience

Participant interview responses related to benefits of the sessions centered around: overall experience, educational materials and reminders, social support and learning from peers, goal setting, the virtual aspect, and self-management (**Table 2**). The overall experience was rated as “good” by most interviewed patients (5/6 patients), and fair by one patient.

**Table 2:** Perceived benefits of structured virtual group-based DSME among patients (N = 6), providers (N = 2), and staff (N = 3).

Theme	Interviewee	Selected quotes
Overall Experience	Patient (Private clinic)	“It's been really great. *I've learned a lot of things that I didn't understand pertaining to being a diabetic*, and how to control, and my feelings and challenges, I've learned a lot.”
	Provider (Private clinic)	“For the providers, at this time are in a difficult time because of lack of access. We have limited time to see patients. We have to finish the visit in a short period of time. *I don't think that providers have the ability to spend the time needed to talk about diabetes, diet, benefits of achieving the goals of managing diabetes, risks of uncontrolled diabetes*. All that in 15-minute, 20 minutes visit, may not be feasible. So having that *support in a group visit can help the physician getting that piece achieved* or done by somebody else, so they can focus on adjusting or managing their medications.”
	Staff (FQHC site)	“That in my experience with the VIDA project, it has been *very rewarding* because I am having the opportunity to make a *significant impact on the life of participants* by teaching the online diabetes classes.” “Because as a registered dietician I had witnessed … firsthand, *the disparities in diabetes education among different populations*, particular minorities like Latinos and African Americans population. And through implementing VIDA project, I feel that I able to start making a little bit of contributions to the disparities by *being part of the team that provide accessible and empowering diabetes education.* And one aspect of my experience with the VIDA project that *stands out is the use of the online classes or online platform*.”
Educational Materials and Reminders	Patient (Private clinic)	“It doesn't matter how long or how short a period you've been diagnosed, *you can always learn something new*.” “…*it reminded me about not only nutrition, but monitoring my blood sugar and how important it can be*, because if I'm doing well, I'm doing good, blood sugar is at a good rate, I have a tendency to not check it, because it's not a very comfortable feeling, pricking your finger … four times a day or so.”
	Provider (Private clinic)	“…we know as providers … that there is evidence that *outreach, communication, connection* with the patient even telephonically can *provide added support in education and encouragement in managing diabetes.*”
	Staff (FQHC site)	“The benefits … is the *education portion for our patients*. We have patients who have either been newly diagnosed with diabetes or those who have had their diagnosis now for a couple of years and have been dealing with their diabetes diagnosis, who have come up and said, "Oh, *this is new information even though I've been diagnosed for 10 years …* this is great to know.”
Social Support and Learning from Peers	Patient (Private clinic)	“Yes, I've learned that others having similar problems like I was, and it was *very informative just to listen to the different things that were happening with other people*, and the same thing was going on.”
	Staff (FQHC site)	“Just having that support and also knowing that there's other patients like them … that are also maybe either struggling with same issues or *hearing from them what they have done, that works best for them so that they can also apply it to their lifestyle*. So I think that's *very beneficial for them just to hear feedback even from people within their own community and within their own health center*.”
Goal Setting	Patient (Private clinic)	“In the class we talked about stress, we talked about nutrition and setting goals … It was *setting goals and sticking and being accountable* for those goals that are set.”
	Provider (Private clinic)	“And getting diabetes under control not only benefit the patient from a health perspective, *it helps the provider achieving the goals*.”
	Staff (FQHC site)	“…very important to clarify that one important thing about our classes is about goal settings. With the goal settings we are teaching participants how to set up SMART goals and *through the SMART goal, we are trying to help them to achieve what they are trying to look in their life* with when management diabetes.”
Virtual Aspect	Patient (FQHC site)	“I think it's a great group to have on Zoom … cause *you can do it right from your home* and the communication, I feel that it's a lot better for people.”
	Provider (Private clinic)	“Virtual visits provide an added benefit in having that personal touch to the patient, where they share their experience, *get the education that is needed without the hassle of driving.*”
	Staff (Private clinic)	“What was helpful about the VIDA study is that because it is *virtual, I think it has really allowed for us to be able to have more regular attendance during the sessions …* the fact that they don't have to commute somewhere to connect to the group calls … I think that if we were to have this be *in-person, they probably wouldn't be able to make it to the sessions right after work* because we start at five o'clock. But because they are conducted virtually, a lot of times they are able to just connect right after work.”
Self-management	Patient (Private clinic)	“Yes, I've learned to speak more with my doctor so that she explains things more explicitly to me, because I've learned that *I am the advocate of myself …* I inform [my doctor] of what's going on. And it seems to be helping because my levels, A1cs, are going down.”
	Provider (FQHC site)	“I think a bigger focus on *lifestyle and diet or food intake and the care of diabetes and not being so focused on the pharmacology …* I think not as much emphasis is placed on everything else, even though it's just as important, so I would hope that it helps the treatment for diabetes, make it more equal, like the lifestyle and the medication, and not just focus so much on the medication.”
	Staff (Private clinic)	“We've seen a lot of situations in our group where some of the successes we have had is that participating in this program has *allowed some patients to really be advocates for their own health …* something that's really great about our group is that *it's become a space where patients start to feel empowered to actually advocate for themselves in their doctor's office …* having those conversations with the [diabetes educator] and other patients as well who have experienced similar situations really encourage that patient to advocate for herself and talk to her physician about things that are not working for her so that she can take on a better role in the management of her own health.”

Italics indicate emphasis added by the authors.

##### Social support

All interviewed patients reported liking the social support aspect of the intervention. Particularly, learning from their peers was a theme that many patients commented on in their interviews: *“Well, you get a chance to talk with other people and see where they are in their journey. Some people are farther along. Some people are just learning about it. You get a chance to share experiences.” - Private health network clinic patient*

##### Educational materials and goal setting

Many patients reported benefiting from the educational materials (curriculum and binder) (5/6 patients) and goal setting provided by the group (6/6 patients). *“My first take on this was, “This is great for someone who is newly diagnosed, who really doesn’t know how to navigate this whole diabetes process.” I remember when I was diagnosed, I was sent to a nutritionist, and then I remember I attended a diabetes management seminar in our local grocery store one evening. They gave me a lot of good information, but other than that, it’s whatever research I could do on my own.” - Private health network clinic patient.*

Patients appreciated the accountability of the group in regard to their SMART goals. One patient from the FQHC said: “*I did have some goals, but I hadn’t expanded upon them until I was asked to.”* Several patients commented on how identifying goals for their diabetes management was helpful to them. A staff member from the private health network clinic stated: *“A lot of our patients … introducing them to SMART goals has allowed them to see that they can create small changes that don’t necessarily have to be extreme lifestyle changes in their life, but that can be short goals that they can set to improve their health little by little…”*

##### Virtual format

Most patients did not comment directly on how beneficial the virtual aspect of the session was. Some patients indirectly mentioned how they appreciated how accessible it was to participate in the session from home. The providers and staff felt the virtual component was important to provide diabetes care to these communities. A private health network clinic provider reflected: “*…some of our patients have difficulty getting to the office or getting access to diabetes clinics or endocrinologists. So we thought that virtual visits are feasible, easy to implement. Patients like it. It doesn’t require additional transportation and the time constraint that most people have.”*

All six patients reported wanting the sessions to continue and stated they would recommend this program to other patients. One FQHC patient responded: “*I would tell them about the [study] group and to see if their provider would offer that for them so that they know that there’s support and help and education behind everything, so that they’ll know that they’re not alone.”*

#### Challenges of structured virtual group-based DSME

##### Participant challenges

Participant interview responses related to challenges of the sessions centered around: overall challenges related to maintaining progress for patients, outreach for project coordinators, keeping track of participants for providers, as well as patient concerns regarding timing and mistrust. While two patients answered that they experienced no challenges, one patient answered that *“getting into technology”* was a challenge and one patient said it was *“hard to maintain”* their progress. Regarding timing of the session, one patient expressed that the late start was a challenge, while another expressed wanting the sessions to start and end on time. Additionally, one patient at the FQHC mentioned privacy and not wanting to have to turn their camera on for the sessions: “*I probably, next time, would not put my camera on. The world is pretty small, so sometimes being diagnosed with something, it’s sort of embarrassing.”*

The significance of having consistent and accurate curriculum material resonated notably through the experiences of two patients who conveyed their mistrust. When expressing their opinion about the provided binder with educational materials, one patient from the FQHC stated: “*Again, I just wanted to make sure, accurate as possible. African-Americans tend to be scared of the medical profession, sorry, almost as much as police, because so many experiments have been done. I want information from two to three different people. So the material … because I can go back … to this book.”* Another patient from the private health network clinic also expressed a need for consistency: *“Now, I’ve heard various opinions about how many carbs you could have … Everybody get on one train … and stop giving conflicting information. Is it 45 to 60 per meal or per day?”*

#### Provider and health system challenges

Both providers reported challenges in receiving updates on their patients participating in the study. Both providers suggested that seeing a progress report or list of the patients participating in the intervention would be beneficial. They mentioned that more staffing is important in order to help provide patients with the information they need outside of a limited 15-minute medical visit. The provider at the private health network clinic explained that a short meeting with the research team to discuss progress among patients would be helpful. The provider at the FQHC mentioned that it would be great to have a visit with their patients after finishing the program to have more continuous care and to be updated on their progress. The FQHC provider stated they found it challenging to keep track of who was actively participating in the study: “*I only spoke to one other patient who seemed to be interested … there hasn’t been enough time for me to check to see if it’s made an impact on their diabetes, so I don’t know too much, honestly, from whether or not my patients were helped or how they were impacted.”*

## Discussion

We conducted a pilot study assessing implementation of virtual group diabetes education sessions at two sites in two distinct healthcare networks in the Chicago metropolitan area. We found that the structured virtual group-based DSME model can be applied in different healthcare systems with different patient populations while remaining consistent to the intervention model and core goals of the diabetes self-management education. Group size was larger in the private site, but that was likely driven by recruitment of six patients relative to only four patients at the FQHC site. Patients declined participation in the virtual group-based DSME sessions most often due to being concerned with other responsibilities and not believing they needed further education. On the other hand, technological concerns were not cited as frequently as a major barrier. Patients from both networks utilized computers and smartphones to log in, highlighting patient access to multiple types of technology which aided delivery of the intervention. Patients at the FQHC had a trend of participating less frequently with their camera on relative to the group in the private clinic. This finding was largely exploratory and would benefit from further exploration with a larger sample size. In qualitative analysis of patient interviews, patient experiences were largely positive with particular appreciation for social support and improved accessibility through the virtual delivery. Key fidelity to intervention metrics like SMART goal creation and discussion of curriculum priorities such as nutrition and exercise were met.

Analyses of provider perspectives on virtual health programs have shown that comfort and access to technological infrastructure are primary concerns with telehealth implementation (21). However, these two barriers were among the least cited by patients in our study as a reason for declining the intervention. This study took place in the Chicago metropolitan area, but many telehealth studies, especially pre-COVID, were designed for rural areas where technological infrastructure is less developed (22). The VIDA project, which started well after the stay-at-home period of the COVID-19 pandemic, may also have been helped by the increase in remote communication which was necessitated by the pandemic. Patients may have been more comfortable and had more access to technology due the uptake of virtual medical visits by health systems. These differences in healthcare context may explain why some of the results depart from current literature.

The positive reception of the virtual education program in this study aligns with prior work demonstrating the efficacy of diabetic self-management interventions. Other interactive virtual models like the LIVE study have shown that technology-enabled self-management education can be successfully implemented in patients with optimal A1c levels in non-Hispanic White individuals (18). We add to this literature by expanding implementation of virtual self-management to a largely African-American population with exclusively suboptimal (>8%) A1c control. This pilot study, unlike the LIVE randomized controlled trial, is not powered to analyze measures of self-management or clinical outcomes. However, this pilot project sets the stage for the upcoming VIDA cluster randomized control trial to analyze whether structured virtual DSME can meaningfully change health outcomes in a population with elevated A1c levels.

Recent studies have proposed that a possible mechanism by which group-based visits improve A1c levels is by reducing diabetes distress, an emotional response to the demands of living with diabetes distinct from depression or anxiety, through group engagement (23). While our pilot study did not examine clinical outcomes like A1c levels, this effect has been demonstrated in multiple systematic reviews (24–27). Our findings highlight the importance of peer encouragement and group support for patients learning diabetes self-management strategies. Many quotes from patients and staff highlight that this group experience improved confidence in advocating for themselves and their health, further tying together the psychological and social dimensions of living with diabetes.

SMART goal setting behaviors have been identified as an effective strategy to significantly reduce A1c levels (28). Our work adds to goal-setting literature by implementing SMART goals within a larger DSME program both virtually and in two, distinct health systems. Across the six sessions, exercise and monitoring diabetes indicators emerged as the most common mentioned topics, reflecting patient priorities related to managing their diabetes. However, when discussing challenges, nutrition was mentioned slightly more than exercise, showing a contrast between aspects of diabetes management that patients struggle with compared to the aspects they are more likely to track, such as their A1c or physical activity. Due to the small sample size, we could not conduct significance testing, but these insights highlight the potential of future research to characterize the potential dichotomy between patient priorities and the behaviors which they are more likely to track via goal-setting.

While the majority of patients reported positive experiences, interviews also highlighted the challenge in cultivating trust with a virtual intervention. Some patients noted that past experiences have made them skeptical of information from medical providers. Patients with distrust of the healthcare system may be more suspicious of information delivered on a virtual platform (29). Further evaluation of these considerations is particularly important given the high prevalence of diabetes within Black and Latino communities, who report higher distrust of healthcare systems (30, 31). Prior conceptual analysis of trust, distrust, and mistrust in medical institutions have described historical factors that contribute to skepticism in medical research (32). One patient in our study specifically cited the history of experimentation on African-American patient populations as a reason for mistrusting the information provided in sessions. This finding is consistent with other studies that have demonstrated the enduring impact of events like the Tuskegee syphilis study on medical mistrust in Black communities (33, 34). However, not all patients ascribed mistrust to historical experiences. Another patient in our study expressed a need for consistency in providing carbohydrate goals. Prior exploration of the barriers towards research participation among Black women identified the importance of a knowledgeable research team, experience with the Black community, and engaged community partners as critical to successful engagement (35). Taken together, the feedback from our study highlights that both historic motives and contemporary competence may be specific factors underpinning distrust or mistrust in medical care. Further recruitment and engagement strategies aimed at building trust with patients, particularly those belonging to marginalized groups, should target the precise mechanism of an individual’s lack of trust in care.

Our study is novel in its attempt to deliver the same structured virtual group-based DSME intervention in both an FQHC and private network. Biweekly cross-system meetings were essential to navigating implementation challenges, improving coordination across study sites, and refining guidance for study staff. One challenge encountered due to the health system differences was that providers from both health networks reported difficulty tracking patient participation. This occurred despite the presence of a notification protocol. This suggests a disconnect between intervention design and real-world workflow integration. It is important to align communication systems with existing clinical processes and it represents a barrier to implementation of virtual DSME. Future studies should further evaluate ways to improve provider awareness and integration of structured virtual group-based DSME participation into routine workflows.

## Limitations

These results were limited by several important factors. The program only utilized two pilot sites, one from each healthcare system, and the total number of patients, 10, was small which hinders generalizability of these findings. This pilot study is exploratory and was not powered to detect modest differences in baseline characteristics between participants in the intervention and those who declined enrollment. Given the reliance of subjective data from site coordinators regarding the reasons for declining participation, there was also an inability to assess the communication skills or strategies utilized by staff that contacted patients for the study. Although the pilot sites differed in terms of healthcare system characteristics, the sites are both located in the Chicago metropolitan area. Rural populations, which utilize telehealth more frequently, were not included, and future structured virtual group-based DSME studies may benefit from implementation in these settings. The sessions and materials were all in English, which limited participation to English-speaking patients. This was a pilot study with limited resources and we did not have the capacity to implement a multilingual intervention but this represents an important limitation, particularly given that the participating FQHC serves a substantial Hispanic/Latino patient population. The exclusion of non-English speaking patients may limit the generalizability of findings while potentially overlooking specific barriers and facilitators to participation. To address this limitation, the upcoming cluster randomized controlled trial has materials and intervention delivery to include Spanish-speaking patients. This limitation is relevant in virtual DSME because language access may influence digital engagement and trust in care delivery. For this pilot project, we conducted qualitative assessment of participant experiences and an assessment of implementation factors, but we did not evaluate clinical outcomes.

## Conclusion

Structured virtual diabetes group-based DSME was implemented in two distinct healthcare settings, an FQHC site and a private, not-for-profit system clinic site. We found that this model was well-liked by providers and patients, with particular appreciation for the social nature of the experience. Although challenges with technology have been documented in telehealth interventions, they were not a major barrier to participation in our study. The utilization of strategies like regular cross-system meetings and structured SMART goal setting helped ensure consistency between the two sites. Future studies should evaluate implementation patterns with larger sample sizes and across a larger number of study sites to improve generalizability. Additionally, more work is necessary to understand how virtual group chronic disease education affects clinical outcomes like A1c, blood pressure, and hospitalization rates in the long term. These findings of this pilot study provide important insights regarding the promise of structured virtual group-based DSME as a vehicle for delivering self-management education for patients with chronic disease.

## Data Availability

The raw data supporting the conclusions of this article will be made available by the authors, without undue reservation.
